# Influence of non-Newtonian Rabinowitsch fluids on the operational efficiency of sine film thrust bearings with porous walls

**DOI:** 10.1038/s41598-025-20308-y

**Published:** 2025-10-21

**Authors:** Deepak Palani, Amit Kumar Rahul

**Affiliations:** Department of Mathematics, School of Advanced Sciences (SAS), Vellore Institute of Technology (VIT), Chennai, 600127 Tamil Nadu India

**Keywords:** Non-Newtonian fluid, Sine film thrust bearing, Rabinowitsch fluid model, Porous wall, Mathematics and computing, Mechanical engineering, Fluid dynamics

## Abstract

This study explores the influence of a porous wall on the performance of a sine film thrust bearing (T-bearing) with the lubrication of non-Newtonian (n-N) Rabinowitsch fluid. Analytical expressions for pressure distribution, load-carrying capacity and volume flow rate are derived using a nonlinear Reynolds equation based on the Rabinowitsch fluid (RF) model, coupled with a small perturbation technique. The findings reveal that dilatant lubricants generate higher peak pressures, enhanced load-carrying capacities and increased volume flow rates compared to Newtonian and pseudo-plastic lubricants. However, an increase in porous pad thickness and capillary tube radius leads to a reduction in these performance metrics. The results underscore the effectiveness of n-N dilatant lubricants in optimizing the performance of T-bearings.

## Introduction

Non-Newtonian rheology examines the behavior of n-N fluids as lubricants under varying stress and strain conditions. Unlike Newtonian fluids, which maintain a constant viscosity, n-N fluids change their viscosity in response to shear rate, influencing factors such as wear, lubrication and friction. Sine thrust bearings, which are precision-engineered components designed to withstand axial loads, provide accurate control and measurement of thrust forces. These bearings are vital in applications requiring precision and reliability such as robotics, aerospace and precision manufacturing. Their design minimizes distortion and ensures smooth rotation, thereby enhancing performance and efficiency. Constructed from high-quality materials, sine thrust bearings are essential for supporting axial loads with precision and durability. While many researchers, including Williams^1^, Hamrock^2^, Lin et al.^3^, Lin and Hung^4^ and Talmage and Carpino^5^, have investigated how thrust bearings behave under load with Newtonian lubricants, Bourgina et al.^6^ predicted the performance of lubricating systems with n-N shear-thinning lubricants using a generalized Reynolds equation and a finite element approach. Safar and Shawkit^7^, Saurabh et al.^8^ and Amalraj et al.^9^ have explored the lubrication performance of thrust bearings with Herschel-Bulkley fluids under sinusoidal injection. Lin et al.^10^ showed that non-Newtonian Rabinowitsch fluids significantly affect the performance of sine film thrust bearings, where dilatant fluids enhance load capacity and friction, while pseudo-plastic fluids reduce them, providing insights for optimized bearing design. Lin^11^ demonstrated that sine film profile thrust bearings achieve higher pressure and load capacity with lower flow rates compared to inclined plane bearings, improving bearing design. Additionally, Shukla et al.^12^ investigated the effect of a sine-shaped film profile on the lubrication of rough thrust bearings using magnetic fluids. The flow characteristics of these n-N lubricants demonstrate a nonlinear correlation between shear stress and shear strain rate. According to Wada and Hayashi^13^ experimental investigations, this non-linear (cubic) relationship is represented by an empirical cubic stress model called the RF model. The cubic correlation between shear stress ${\bar {\tau }_{xy}}$ and strain rate $\frac{{\partial \bar {u}}}{{\partial \bar {y}}}$ is given by:

1 $${\bar {\tau }_{xy}}+\bar {k}{\bar {\tau }_{xy}}^{3}={\bar {\mu }_0}\frac{{\partial \bar {u}}}{{\partial \bar {y}}}$$

In this context, ${\bar {\mu }_0}$​ represents the initial viscosity of the Newtonian fluid, while $\bar {k}$ is the nonlinear component that characterizes the n-N fluid properties. The flow behavior can be categorized into three types: $\bar {k}=0$ corresponds to a Newtonian fluid with constant viscosity, $\bar {k}<0$ indicates shear-thickening (dilatant) behavior and $\bar {k}>0$ represents shear-thinning (pseudoplastic) behavior, as described by Wada et al.^13^. Several researchers have extensively studied thrust bearings, with a particular focus on applying the RF model to enhance their performance. Singh et al.^14^ analyzed the impact of n-N shear-thinning lubricants and lubricant inertia on annular hydrostatic thrust bearings, while Lin et al.^15^ employed the RF model with a nonlinear Reynolds equation to evaluate n-N lubricants in sliding bearings. The impact of variations in viscosity in n-N fluids on the performance of conical bearings with porous walls has been studied by Rao and Rahul^16^ and examined the influence of surface roughness and viscosity changes on rough conical bearings using n-N Rabinowitsch fluids. Additionally, Singh^17^ analyzed the effects of surface roughness and lubricant inertia on the operation of stepped circular hydrostatic thrust bearings using n-N Rabinowitsch fluids, emphasizing the critical role of lubricant films in preventing direct contact between mating surfaces and the importance of bearing surface porosity in retaining lubricants, thereby reducing lubrication frequency and enhancing performance.

The small perturbation method is a widely used analytical technique in lubrication theory and fluid mechanics for simplifying complex nonlinear problems that involve small deviations from an undisturbed state. It assumes that the perturbation effect is minor relative to the primary flow behavior. Therefore, variables such as velocity and pressure are expanded in a power series of a small, dimensionless parameter. By substituting these expansions into the governing equations and neglecting higher-order terms, the resulting system becomes linearized and easier to solve, as outlined by Pai and Luo^18^. This method enables accurate approximation of system behavior while preserving physical insights and is particularly effective in thin-film lubrication problems. Penesis et al.^19^ used it to analyze gas-lubricated slider bearings, while recent works by Abbaspur et al.^20^ and Pfeil et al.^21^ applied perturbation-based techniques to nonlinear viscoelastic lubrication and semi-analytical Reynolds equation solutions, respectively.

Porous bearings, which are widely used in engineering due to their ability to retain lubricants, reduce lubrication frequency, low cost, and independence from external oil supply, have been the focus of numerous studies. An additional critical design factor in thrust bearings is the use of porous walls, which improve lubricant retention, reduce external oil supply requirements, and promote thermal stability. Early work by Morgan and Cameron^22^ employed Darcy’s law to model flow through porous media, later modified for non-Newtonian behavior by Wada et al.^23^. Modern studies have expanded on this by analyzing the performance of porous structures in various bearing configurations. Recent literature on porous thrust bearings underscores the continued focus on performance enhancement through structural design innovations. Kumar et al.^24^ investigated the dynamic behavior of externally pressurized porous thrust bearings with varying pocket shapes, demonstrating how geometric configurations influence load capacity and stability. Sahto et al.^25^ analyzed the effect of partial orifice porosity in aerostatic thrust bearings, highlighting improvements in stiffness and damping characteristics. Kumar et al.^26^ explored the rotor-dynamic performance of porous hydrostatic thrust bearings subjected to magnetic fields, revealing the interplay between magnetic actuation and porous media in regulating dynamic stability and operational efficiency. Moreover, Munshi et al.^27^ showed that a sine film profile with ferrofluid and roughness enhances load support in slider bearings. Rao and Rahul^28^ examined the influence of viscosity variation and surface roughness in porous conical bearings using Rabinowitsch fluids. Their findings indicate that dilatant fluids provide superior load capacity, while pseudoplastic fluids reduce it due to shear-thinning at high strain rates^29,30^.

The majority of research has focused on the effects of non-Newtonian fluids and porous structures separately, despite these developments. It is still entirely unknown how to combine complex rheological models like the RF model, complicated geometries like sine profiles, and porous media behavior in a single, cohesive method. Addressing this gap is crucial to building next-generation thrust bearings that satisfy the requirements of self-regulating, high-precision, high-load systems in advanced manufacturing, robotics, and aerospace applications.

### Research gap

While several research have examined thrust bearing performance utilizing non-Newtonian fluids or porous materials separately, the combined impact of non-Newtonian Rabinowitsch fluids within porous sine film thrust bearings has received little attention. Prior study has primarily focused on isolated components, such as pressure augmentation from capillary-tube flow in porous media or the nonlinear shear properties of non-Newtonian fluids.

However, the interacting consequences of these mechanisms—particularly in the configuration of sinusoidal film geometries and variable structural factors such as inlet-to-outlet film thickness ratios, porous pad thickness, and capillary tube dimensions—have not been thoroughly investigated. Furthermore, the effects of certain non-Newtonian fluid behaviors—specifically, dilatant (shear-thickening) and pseudoplastic (shear-thinning) properties—on the functionality of porous thrust bearings are not well understood. This includes how they interact with porous bearing walls and how that affects critical measurements of performance including load-carrying capacity, pressure distribution, and volume flow rate.

Recent developments in porous thrust bearing design, such as studies of pocket geometries, orifice configurations, and the impact of magnetic fields, have not yet been investigated in the context of Rabinowitsch-type non-Newtonian fluids. This gap emphasizes the importance of an integrated approach to understanding the interaction of advanced non-Newtonian fluid models and porous media effects in thrust bearing systems. Addressing this will provide new insights that are essential for the design of high-efficiency, low-maintenance, and dependable bearing systems, particularly for precision engineering applications.

### Novelty

By considering the combined effects of porous wall and non-Newtonian Rabinowitsch fluid behavior—an area that has not received much attention in prior research—this study creates a novel mathematical framework for evaluating sine film thrust bearings. This work generates a nonlinear modified Reynolds equation for flow through porous media that considers Darcy’s law and the Rabinowitsch model, in contrast to prior analyses that analyze fluid rheology and porous effects independently. The modeling of complex fluid-structure interactions inside the lubricant coating and porous substrate is made possible by this comprehensive formulation.

The use of the small perturbation method to obtain closed-form analytical expressions for critical performance parameters including volumetric flow rate, pressure distribution, and load-carrying capacity is a significant innovation for porous thrust bearing. This study also provides a comprehensive comparative analysis of dilatant, Newtonian, and pseudoplastic fluids, demonstrating how variations in porous design parameters—such as pad thickness and capillary tube radius—significantly affect bearing performance. By integrating non-Newtonian rheology with porous media effects under sinusoidal film geometries, this research advances the theoretical understanding of complex lubrication systems. The insights gained lay the foundation for improved design and performance optimization of high-precision thrust bearings in advanced mechanical and tribological applications.

### Objective

- The primary goal of this study is to improve the operational performance of sine film thrust (T-) bearings by developing a comprehensive analytical framework that accounts for the combined effects of porous wall structures and non-Newtonian Rabinowitsch fluid behavior. Moreover, this study aims to derive a nonlinear Reynolds equation by incorporating the Rabinowitsch fluid model with a modified form of Darcy’s law, thereby enabling accurate prediction of essential lubrication characteristics such as load-carrying capacity, pressure distribution, and volumetric flow rate.
- To study the film geometry, porous pad thickness, and capillary tube radius affect bearing system performance, particularly under different non-Newtonian regimes, such as dilatant (shear-thickening) and pseudoplastic (shear-thinning) fluid responses. In doing so, the study hopes to uncover the complex interplay between fluid rheology and porous media effects.
- To develop strong design guidelines for advanced thrust bearing systems, with the goal of improving efficiency, reducing wear and maintenance, and increasing operational stability under complex and demanding conditions.

## Governing equations and boundary conditions

**Fig. 1:**
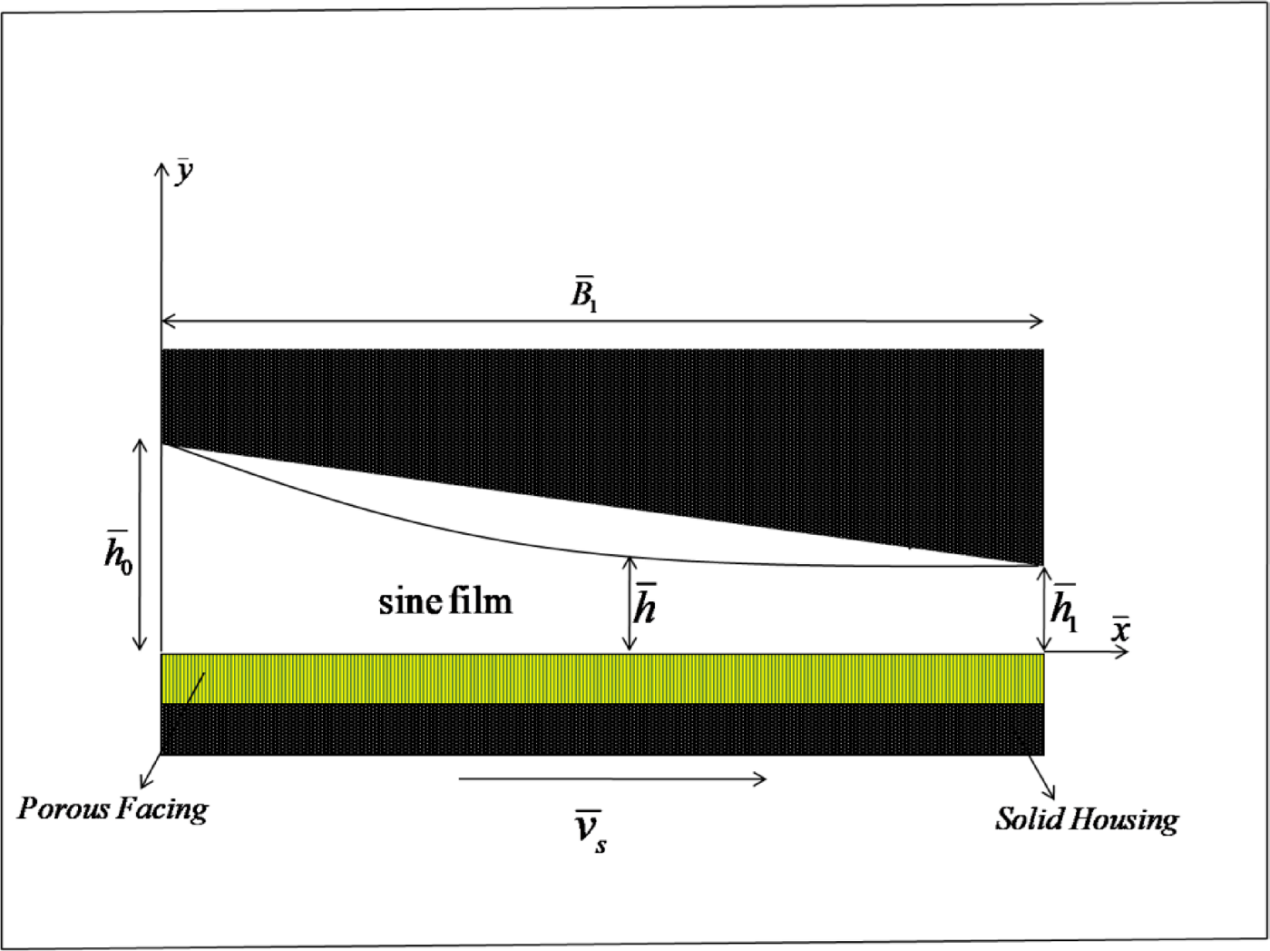
Physical geometry of a sine film porous thrust bearing lubricated with n-N fluid.

Figure 1 depicts the physical configuration of a sine film T-bearing lubricated with an n-N fluid. The bearing has a length in the $\bar {x}$-direction and a width ${\bar {B}_1}$ perpendicular to the $\bar {x}\,\bar {z}$-plane. In this study, the bearing is assumed to be infinitely wide, meaning ${\bar {B}_2}$ is much greater than ${\bar {B}_1}$. The film height at the inlet along the $\bar {y}$-direction is ${\bar {h}_0}$​, while at the outlet, it is ${\bar {h}_1}$​. The $\bar {x}$-direction sliding velocity is represented by ${\bar {v}_s}$. The equation describing the local film height for the sine film bearing is as follows:

2 $$\bar {h}(\bar {x})={\bar {h}_1}+{\bar {h}_{\sin }}(\bar {x})$$

Where ${\bar {h}_0}$ and ${\bar {h}_1}$ represent the inlet film height and outlet film height, the sine curve ${\bar {h}_{\sin }}(\bar {x})$ can be represented as:

3 $${\bar {h}_{\sin }}(\bar {x})=({\bar {h}_0} - {\bar {h}_1})\left[ {1 - sin\left( {\frac{{\pi \bar {x}}}{{2{{\bar {B}}_1}}}} \right)} \right]$$

Assuming a very low Reynolds number $({\text{Re}} \ll 0)$, consistent with thin-film lubrication theory, the flow is considered incompressible, and inertia terms are neglected. Under these assumptions, the continuity and momentum equations simplify as follows, as described by Hamrock^2^:

4 $$\frac{{\partial \bar {u}}}{{\partial \bar {x}}}+\frac{{\partial \bar {v}}}{{\partial \bar {y}}}=0$$

5 $$\frac{{\partial {{\bar {\tau }}_{xy}}}}{{\partial \bar {y}}}=\frac{{\partial \bar {p}}}{{\partial \bar {x}}}$$

6 $$\frac{{\partial \bar {p}}}{{\partial \bar {y}}}=0$$

Where $\bar {u}$ and $\bar {v}$ represent the velocity components in the $\bar {x}$ and $\bar {y}$ directions respectively, and $\bar {p}$ denotes the film pressure.

The boundary conditions in the $\bar {x}$-direction are:

7 $$\bar {y}=0 \; at \; \bar {u}={\bar {v}_s}$$

8 $$\bar {y}=\bar {h} \; at \; \bar {u}=0$$

## Mathematical formulation and solution

The expression for the velocity component can be obtained by solving Eqs. (1) and (5) together with the pertinent boundary conditions.

9 $$\bar {u}={\bar {u}_1}+{\bar {u}_2}$$

10 $${\bar {u}_1}=\frac{1}{{{{\bar {\mu }}_0}}}\left[ {\frac{1}{2}\left( {{{\bar {y}}^2} - \bar {h}\bar {y}} \right)\left( {\frac{{\partial \bar {p}}}{{\partial \bar {x}}}} \right)+\bar {k}\left[ {\frac{{{{\bar {y}}^4}}}{4} - \frac{1}{2}{{\bar {y}}^3}\bar {h}+\frac{3}{8}{{\bar {y}}^2}{{\bar {h}}^2} - \frac{1}{8}\bar {y}{{\bar {h}}^3}} \right]{{\left( {\frac{{\partial \bar {p}}}{{\partial \bar {x}}}} \right)}^3}} \right]$$

11 $${\bar {u}_2}={\bar {v}_s} - \frac{{{{\bar {v}}_s}}}{{\bar {h}\left\{ {1+\bar {k}{{\left( {\frac{{\bar {h}}}{2}\frac{{\partial \bar {p}}}{{\partial \bar {x}}}} \right)}^2}} \right\}}}\left( {\bar {y}+\bar {k}\left( {{{\bar {y}}^3} - \frac{3}{2}{{\bar {y}}^2}\bar {h}+\frac{3}{4}\bar {y}{{\bar {h}}^2}} \right){{\left( {\frac{{\partial \bar {p}}}{{\partial \bar {x}}}} \right)}^2}} \right)$$

Under the $\bar {y}$-direction, the following velocity boundary conditions are:

12 $$\bar {y}=0\,at\,\bar {v}={w_{prs}}$$

13 $$\bar {y}=\bar {h}\,at\,\bar {v}=\frac{{\partial \bar {h}}}{{\partial \bar {t}}}$$

Equation (4) for continuity, when integrated over the local film height:

14 $$\int\limits_{0}^{{\bar {h}}} {\,\frac{{\partial \bar {u}}}{{\partial \bar {x}}}} \,d\bar {y}+\int\limits_{0}^{{\bar {h}}} {\,\frac{{\partial \bar {v}}}{{\partial \bar {y}}}\,} d\bar {y}=0$$

By applying the corresponding boundary conditions, for the sine film bearing, a nonlinear n-N Reynolds equation can be obtained.

15 $$\frac{\partial }{{\partial \bar {x}}}\left\{ {\frac{1}{{{{\bar {\mu }}_0}}}\left[ {{{\bar {h}}^3}\left( {\frac{{\partial \bar {p}}}{{\partial \bar {x}}}} \right)+\frac{3}{{20}}\bar {k}\,{{\bar {h}}^5}{{\left( {\frac{{\partial \bar {p}}}{{\partial \bar {x}}}} \right)}^3}} \right]} \right\}=12{w_{prs}} - 12\frac{{\partial {{\bar {h}}_{\sin }}\left( {\bar {x}} \right)}}{{\partial \bar {t}}}+6{\bar {v}_s}\frac{{\partial {{\bar {h}}_{\sin }}\left( {\bar {x}} \right)}}{{\partial \bar {x}}}$$

The RF flow in a porous medium is evaluated using a modified Darcy’s law^23^. These homogeneous, isotropic porous layers comprise a network of capillaries with an average radius ${r_c}$ and porosity $\phi$. At the porous wall, the velocity components in the axial and radial directions are

16 $${u_{prs}}=\frac{\phi }{{{{\bar {\mu }}_0}}}\left( { - \frac{{\partial p}}{{\partial \bar {x}}}} \right)+\frac{\phi }{{{{\bar {\mu }}_0}}}\frac{{\bar {k}{r_c}^{2}}}{6}{\left( { - \frac{{\partial p}}{{\partial \bar {x}}}} \right)^3}$$

17 $${w_{prs}}=\frac{\phi }{{{{\bar {\mu }}_0}}}\left( { - \frac{{\partial p}}{{\partial \bar {y}}}} \right)+\frac{\phi }{{{{\bar {\mu }}_0}}}\frac{{\bar {k}{r_c}^{2}}}{6}{\left( { - \frac{{\partial p}}{{\partial \bar {y}}}} \right)^3}$$

Where ${\phi _p}$ is the porosity coefficient, $\phi =\frac{{{\phi _p}{r_c}^{2}}}{8}$ is the permeability of the porous layer, and ${u_{prs}}\& {w_{prs}}$ are the velocity elements in the porous layer. By inserting Eq. (17) into the Reynolds-type Eq. (15), the modified Reynolds equation can be derived as follows:

18 $$\begin{gathered} \frac{\partial }{{\partial \overset{\lower0.5em\hbox{$\smash{\scriptscriptstyle\rightharpoonup}$}} {x} }}\left\{ {\frac{1}{{{{\bar {\mu }}_0}}}\left[ {{{\bar {h}}^3}\left( {\frac{{\partial \bar {p}}}{{\partial \bar {x}}}} \right)+\frac{3}{{20}}\bar {k}\,{{\bar {h}}^5}{{\left( {\frac{{\partial \bar {p}}}{{\partial \bar {x}}}} \right)}^3}} \right]} \right\}=12\left( {\frac{\phi }{{{{\bar {\mu }}_0}}}{{\left[ {\left( { - \frac{{\partial p}}{{\partial \bar {y}}}} \right)+\frac{{\bar {k}{r_c}^{2}}}{6}{{\left( { - \frac{{\partial p}}{{\partial \bar {y}}}} \right)}^3}} \right]}_{\bar {y}=0}} - \frac{{\partial {{\bar {h}}_{\sin }}\left( {\bar {x}} \right)}}{{\partial \bar {t}}}} \right)+6{{\bar {v}}_s}\frac{{\partial {{\bar {h}}_{\sin }}\left( {\bar {x}} \right)}}{{\partial \bar {x}}} \hfill \\ \end{gathered}$$

19 $${\left[ {\left( { - \frac{{\partial p}}{{\partial \bar {y}}}} \right)+\frac{{\bar {k}{r_c}^{2}}}{6}{{\left( { - \frac{{\partial p}}{{\partial \bar {y}}}} \right)}^3}} \right]_{\bar {y}=0}}= - \overline {{{H_p}}} \frac{\partial }{{\partial \bar {x}}}\left\{ {\left( { - \frac{{\partial p}}{{\partial \bar {x}}}} \right)+\frac{{\bar {k}{r_c}^{2}}}{6}{{\left( { - \frac{{\partial p}}{{\partial \bar {x}}}} \right)}^3}} \right\}$$

Using the Morgan-Cameron approximation, the averaged film pressure for the porous wall can be used to replace the film pressure in the porous region^22^. Equation (19) was substituted into Eq. (18) to modify the non-linear Reynolds equation:

20 $$\frac{\partial }{{\partial \bar {x}}}\left\{ {\left[ {\left( {{{\bar {h}}^3} - \frac{3}{2}\overline {{{H_p}}} {\phi _p}{r_c}^{2}} \right)\left( {\frac{{\partial \bar {p}}}{{\partial \bar {x}}}} \right)+\frac{3}{{20}}\bar {k}\left( {{{\bar {h}}^5} - \frac{5}{3}\overline {{{H_p}}} {\phi _p}{r_c}^{4}} \right){{\left( {\frac{{\partial \bar {p}}}{{\partial \bar {x}}}} \right)}^3}} \right]} \right\}=6{\bar {\mu }_0}{\bar {v}_s}\frac{{\partial {{\bar {h}}_{\sin }}\left( {\bar {x}} \right)}}{{\partial \bar {x}}}$$

The modified Reynolds equation is crucial for evaluating the influence of n-N fluid properties and porosity on the film pressure in a sine film T-bearing. Understanding these factors enables the determination of the bearings pressure, load-carrying capacity and flow rate. To effectively analyze bearing performance, the following parameters and non-dimensional variables are mentioned below:

21 $$\begin{gathered} X=\frac{{\bar {x}}}{{{{\bar {B}}_1}}},\,\,\,\,H=\frac{{\bar {h}}}{{{{\bar {h}}_1}}},\,\,\,\,\,{P^*}=\frac{{\bar {p}{{\bar {h}}_1}^{2}}}{{{{\bar {\mu }}_0}{{\bar {v}}_s}{{\bar {B}}_1}}},\,\,\,\,\,R=\frac{{{{\bar {h}}_0}}}{{{{\bar {h}}_1}}},\, \hfill \\ K=\bar {k}\frac{{{{\bar {\mu }}_0}^{2}{{\bar {v}}_s}^{2}}}{{{{\bar {h}}_1}^{2}}},\,\,\,\,\,{H_{Sin}}=\frac{{{{\bar {h}}_{\sin }}\left( {\bar {x}} \right)}}{{{{\bar {h}}_1}}},\,\,\,\,\,{H_p}=\frac{{\overline {{{H_p}}} {\phi _p}}}{{{{\bar {h}}_1}}},\,\,\,\,\,{R_p}=\frac{{{r_c}}}{{{{\bar {h}}_1}}}.\, \hfill \\ \end{gathered}$$

Now, dimensionless film height for the sine film Profile can be represented from Eqs. (2) and (3) are:

22 $$H=1+{H_{\sin }}$$

23 $${H_{\sin }}=\frac{{{{\bar {h}}_{\sin }}}}{{{{\bar {h}}_1}}}=(R - 1)\left[ {1 - \sin \left( {\frac{{\pi X}}{2}} \right)} \right]$$

Thus, by applying the non-dimensional variables and parameters from Eq. (21) to Eq. (20), the non-dimensional modified Reynolds equation can be obtained as follows:

24 $$\frac{\partial }{{\partial X}}\left\{ {\left[ {20\left( {{H^3} - \frac{3}{2}{H_p}{R_p}^{2}} \right)\left( {\frac{{\partial {P^*}}}{{\partial X}}} \right)+3K\left( {{H^5} - \frac{5}{3}{H_p}{R_p}^{4}} \right){{\left( {\frac{{\partial {P^*}}}{{\partial X}}} \right)}^3}} \right]} \right\}=120\frac{{\partial {H_{\sin }}}}{{\partial X}}$$

### Film pressure

By applying the small perturbation method to the film pressure for small values of the nonlinear n-N parameter in $K=0$ Newtonian fluids, as well as in $K>0$ shear- thinning (pseudoplastic) and $K<0$ shear- thickening (dilatant) fluids, the analysis can be carried out as follows:

25 $${P^*}={P_0}^{*}+K{P_1}^{*}+o({K^2})$$

By substituting this expansion into the non-dimensional Reynolds Eq. (24) and collecting terms of order $o({K^0})$ and $o({K^1})$, we derive two coupled linear equations that govern the zero-order and first-order pressures, respectively.

26 $$\frac{\partial }{{\partial X}}\left\{ {({H^3} - \frac{3}{2}{H_p}{R_p}^{2})\left( {\frac{{\partial {P_0}^{*}}}{{\partial X}}} \right)} \right\}= - 3(R - 1)\pi \cos \left( {\frac{{\pi X}}{2}} \right)$$

27 $$\frac{\partial }{{\partial X}}\left\{ {20\left( {{H^3} - \frac{3}{2}{H_p}{R_p}^{2}} \right)\left( {\frac{{\partial {P_1}^{*}}}{{\partial X}}} \right)+3\left( {{H^5} - \frac{5}{3}{H_p}{R_p}^{4}} \right){{\left( {\frac{{\partial {P_0}^{*}}}{{\partial X}}} \right)}^3}} \right\}=0$$

The boundary conditions for the pressure in the film are:

28 $${P_0}^{*}=0\,\,\,\,\,{\text{at}}\,\,\,\,\,X=0\,\,{\text{and}}\,\,X=1$$

29 $${P_1}^{*}=0\,\,\,\,\,{\text{at}}\,\,\,\,\,X=0\,\,{\text{and}}\,\,X=1$$

By solving these two differential Eqs. (26) and (27) and applying the relevant boundary conditions, the pressure result can be obtained:

30 $${P^*}=\frac{{6({f_{21}}{f_1} - {f_{11}}{f_2})}}{{{f_{21}}}}+K\frac{{162({f_{31}} - {f_3})}}{5}$$

31 $${f_1}=\int\limits_{{X=0}}^{X} {\frac{{{H_{\sin }}}}{{{g_1}}}\,dX}$$

32 $${f_2}=\int\limits_{{X=0}}^{X} {\frac{1}{{{g_1}}}\,dX}$$

33 $${f_{11}}=\int\limits_{{X=0}}^{1} {\frac{{{H_{\sin }}}}{{{g_1}}}\,dX}$$

34 $${f_{21}}=\int\limits_{{X=0}}^{1} {\frac{1}{{{g_1}}}\,dX}$$

35 $${f_3}=\int\limits_{{X=0}}^{X} {\frac{{{g_2}\,{s^3}}}{{g_{1}^{4}}}dX}$$

36 $${f_{31}}=\int\limits_{{X=0}}^{1} {\frac{{{g_2}\,{s^3}}}{{g_{1}^{4}}}dX}$$

37 $$s=\frac{{{H_{\sin }}\,{f_{21}} - {f_{11}}}}{{{f_{21}}}}$$

38 $${g_1}={H^3} - \frac{3}{2}{H_p}{R_p}^{2}$$

39 $${g_2}={H^5} - \frac{5}{3}{H_p}{R_p}^{4}$$

### Load-carrying capacity

By integrating the pressure of the film, the load-carrying capacity ($\bar {w}$), can be calculated.

40 $$\bar {w}=\int\limits_{0}^{{{{\bar {B}}_1}}} {\bar {p}\,{{\bar {B}}_2}} d\bar {x}$$

Present the dimensionless load-carrying capacity $({W^*})$as follows:

41 $${W^*}=\frac{{\bar {w}{{\bar {h}}_1}^{2}}}{{{{\bar {\mu }}_0}{{\bar {v}}_s}{{\bar {B}}_1}{{\bar {B}}_2}}}$$

After completing the integration, the non-dimensional load-carrying capacity is obtained:

42 $${W^*}=\int\limits_{0}^{1} {{P^*}} \,dX$$

### Volume flow rate

The required volume flow rate $\bar {q}$ can be obtained by integrating the velocity field.

43 $$\bar {q}=\int\limits_{0}^{{\bar {h}}} {\bar {u}\,} {\bar {B}_1}\,d\bar {y}$$

The non-dimensional flow rate is introduced as follows:

44 $${Q^*}=\frac{{\bar {q}}}{{{{\bar {v}}_s}{{\bar {h}}_1}{{\bar {B}}_2}}}$$

The non-dimensional volume flow rate $({Q^*})$ is obtained after performing the integration.

45 $${Q^*}=\frac{H}{2} - \frac{{{H^3}}}{{12}}\left( {\frac{{\partial {P^*}}}{{\partial X}}} \right) - K\frac{{{H^5}}}{{80}}{\left( {\frac{{\partial {P^*}}}{{\partial X}}} \right)^3}$$

## Result and discussion

The present work focuses on how sine film T-bearings with porous walls perform in the terms of lubrication when n-N Rabinowitsch fluids are used. It relies on a nonlinear Reynolds equation derived from the RF model to produce analytical formulations predicting load-carrying capacity, pressure distribution and volume flow rate. For this special case, the value $K \to 0$ :

46 $$\frac{\partial }{{\partial X}}\left\{ {\left[ {20\left( {{H^3} - \frac{3}{2}{H_p}{R_p}^{2}} \right)\left( {\frac{{\partial {P^*}}}{{\partial X}}} \right)} \right]} \right\}=120\frac{{\partial {H_{\sin }}}}{{\partial X}}$$

Moreover, the study presents the pressure distribution $({P^*})$ for various inlet-outlet film ratios $(R)$, film thickness $(H)$, dimensionless porous pad thickness $({H_P})$​ and capillary tube radius $({R_P})$ as a function of the coordinate $(X)$considering dilatant, Newtonian and pseudo-plastic lubricants. The load carrying capacity $({W^*})$ is shown relative to $(H)$ for varying *R*, ${R_P}$​ and ${H_P}$​. The volume flow rate $({Q^*})$ is analyzed against *X* for a fixed *R*, highlighting the impact of ${R_P}$​ and ${H_P}$​ with n-N dilatant lubricants

### Film pressure

**Fig. 2:**
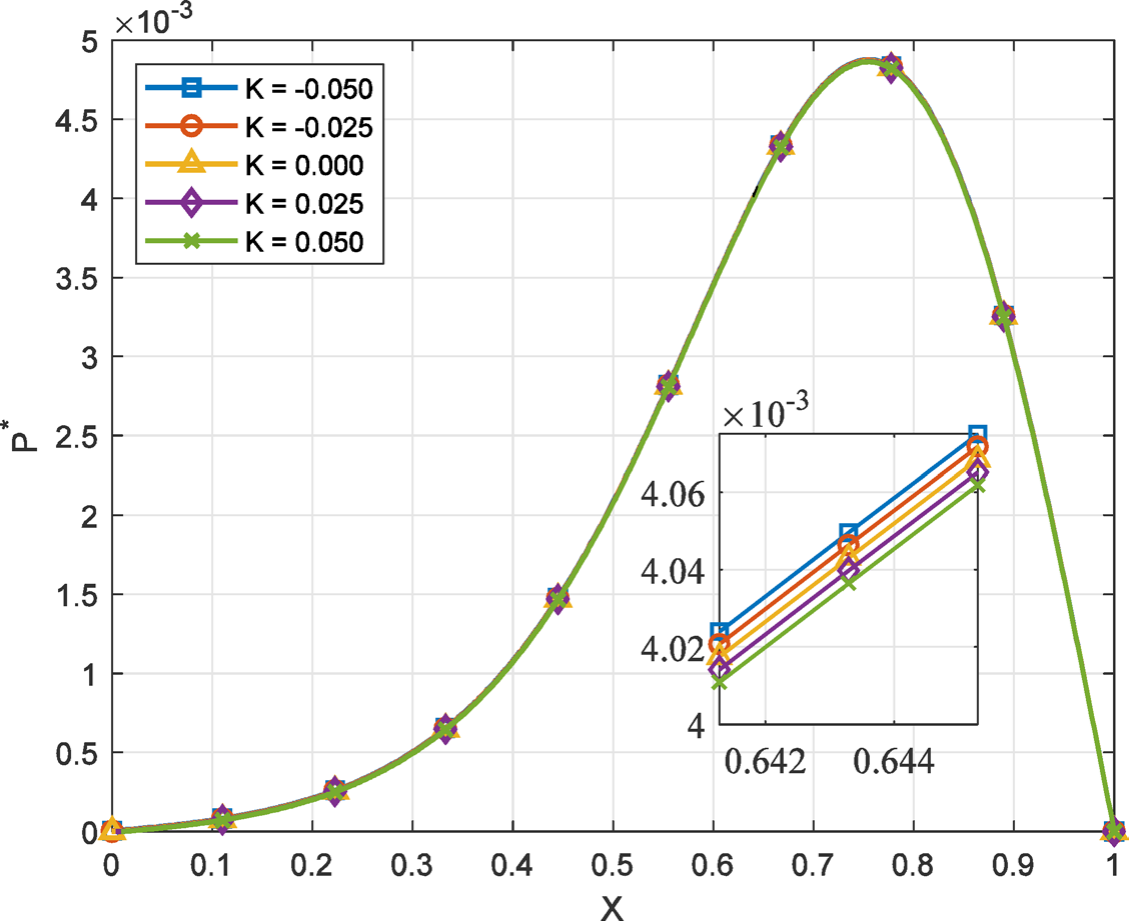
Variation of film pressure $({P^*})$ along the coordinate axis *X*, influenced by ${H_P}$,${R_P}$, *R* for different values *K*.

The main features of the film pressure distribution of the sine film T-bearing with coordinate axis (X) under the influence of the inlet-outlet film ratiosand porosity, for the various values of non-Newtonian parameter is shown in Fig. 2.The pressure rises from the inlet, reaches a peak near the mid-region, and then decreases toward the outlet. This general pressure behavior is similar for all values of K, but the shape and sharpness of the curve are influenced by the fluid’s shear response. For dilatant fluids(K<0), which exhibit shear-thickening behavior, the resistance to flow increases as the shear rate increases. This increased resistance leads to a steeper pressure gradient within the film, particularly near regions of rapid variation in film thickness.

The interaction between the fluid’s shear-thickening nature, the pressure gradient, and the porous flow plays a key role in the overall behavior. In this case, the increased resistance tends to reduce the flow through the porous pad ${H_P}$ and capillary tube ${R_P}$​, which helps maintain higher internal pressure along the bearing surface. In contrast, pseudo-plastic fluids $(K>0)$show reduced resistance at higher shear rates, allowing smoother flow and resulting in a more evenly distributed pressure profile. The inlet-outlet pressure ratio *R* further affects how pressure is sustained and balanced across the bearing surface. Even small changes in *K* result in noticeable differences in the pressure distribution, indicating the high sensitivity of the system to non-Newtonian effects. The combined influence of shear behavior, sinusoidal geometry, and flow through porous and capillary elements governs the load-supporting characteristics of the thrust bearing. These results highlight the importance of considering non-Newtonian properties in the design and analysis of such lubrication systems.

**Fig. 3:**
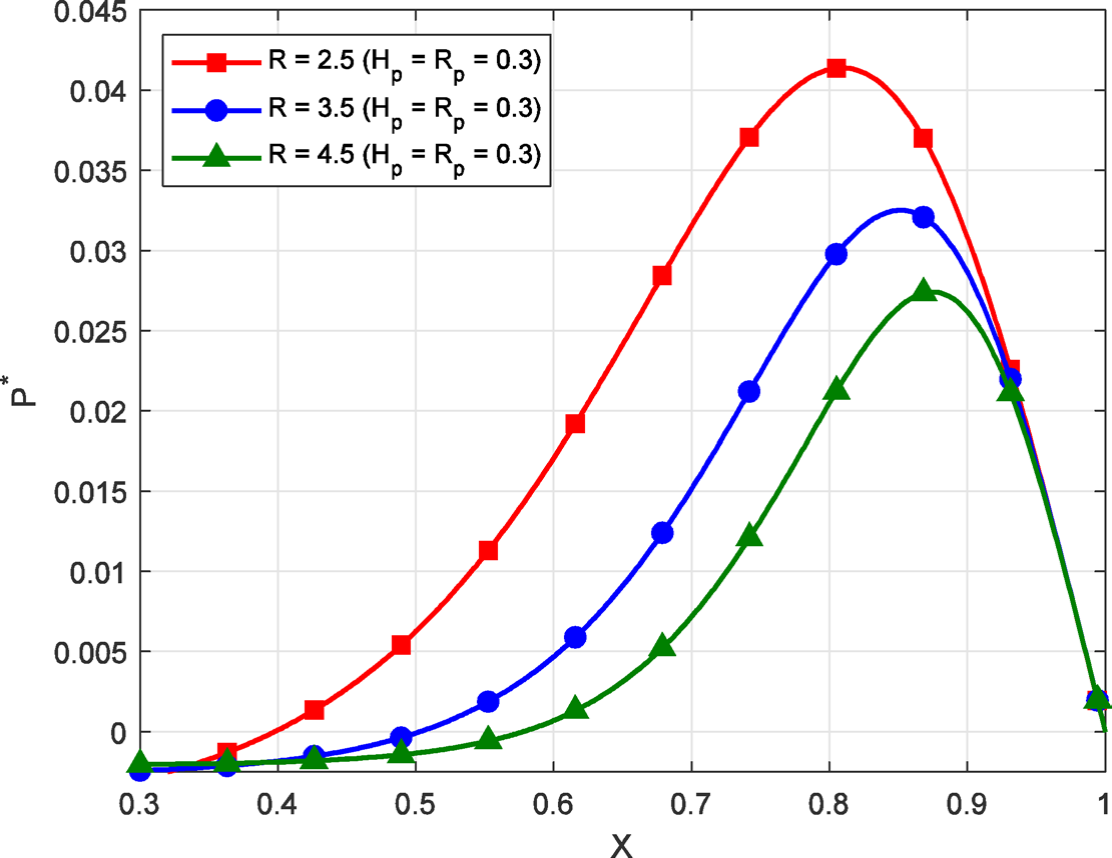
Variation of film pressure $({P^*})$ along the coordinate axis *X*, influenced by ${H_P}$,${R_P}$, *K* for different values *R*.

Figure 3 illustrates the variation of non-dimensional film pressure $({P^*})$ along the coordinate axis *X* for different values of the inlet-to-outlet pressure ratio$(R)$, with constant porous${H_P}\,,\,\,{R_P}=0.3$. The results show that as *R* increases from $2.5{\text{ to }}4.5$, the film pressure decreases across the profile, and the pressure peak becomes smaller. The behavior occurs because a higher *R* value corresponds to a higher inlet pressure relative to the outlet. Although it might suggest more pressure is available at the entrance, the system must still maintain continuity and balance through the porous and capillary zones. When *R *is large, the fluid tends to escape more easily through the outlet, reducing pressure retention along the film. As a result, the internal pressure build-up within the lubricating film reduces, leading to a lower pressure profile. In contrast, a smaller *R* implies a more balanced or moderate inlet-to-outlet pressure condition, which restricts excessive outflow and supports stronger pressure development within the film. This also promotes a more effective interaction between the film pressure and the porous structure, allowing fluid to be retained longer and contributing to higher load support. Overall, the fixed values of ${H_P}$ and ${R_P}$ ​ ensure that the observed differences are primarily due to changes in the boundary pressure ratio *R*. The interaction between inlet-outlet ratio and the flow through the porous and capillary regions plays a key role in shaping the pressure distribution. These findings emphasize the importance of carefully selecting *R* in order to control the pressure gradient and achieve desirable performance in non-Newtonian lubricated thrust bearings.

**Fig. 4:**
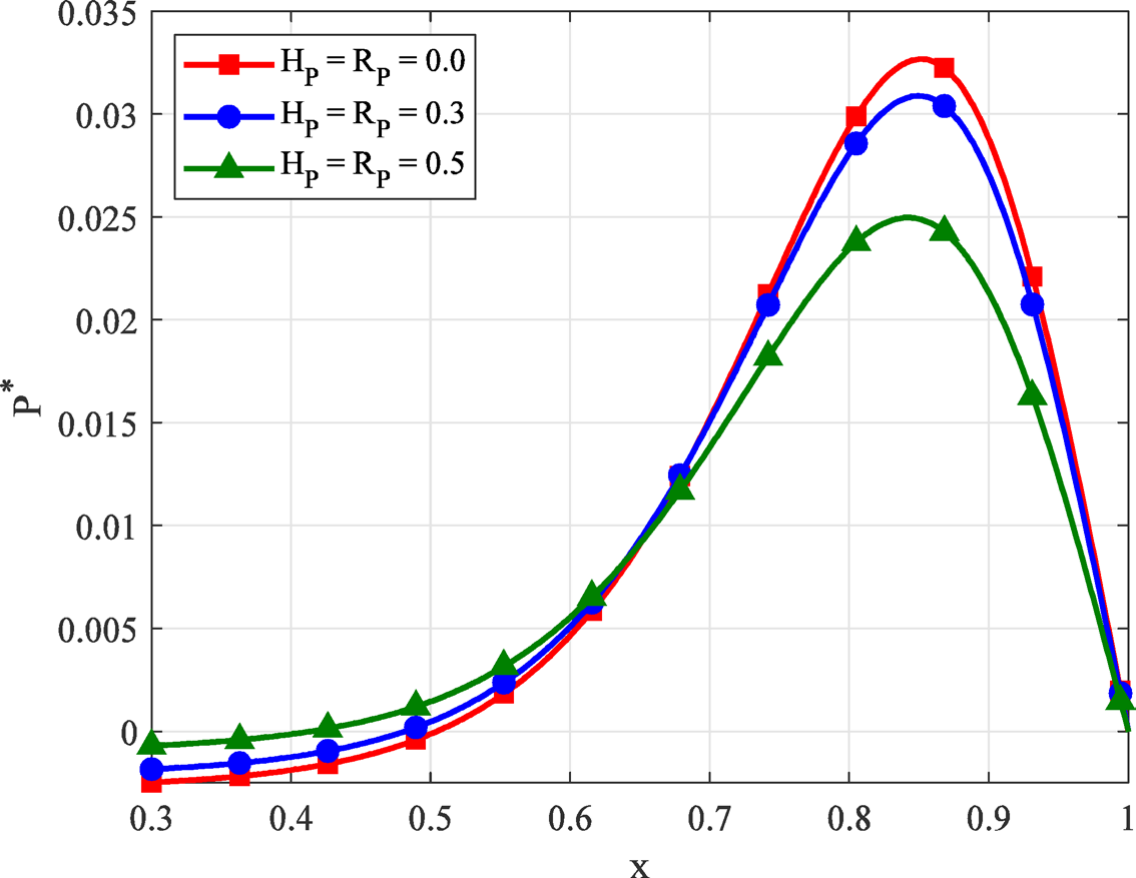
Variation of film pressure $({P^*})$ along the coordinate axis *X*, with *R*, *K* for different values ${H_P}$, ${R_P}$.

Figure 4 illustrates the variation of the film pressure $({P^*})$ along the axial coordinate *X* for different porous parameter ${H_P}\,,\,\,{R_P}=0.0,\,0.3,\,0.5$, while keeping *R* and *K* constant. The results clearly indicate that the peak film pressure diminishes as the porous parameters ${H_P}$ and ${R_P}$ increase. When porosity is absent $({H_P}\,,\,\,{R_P}=0.0)$, the system sustains the highest pressure buildup due to minimal fluid leakage through the pad. However, as porosity increases, more fluid escapes into the porous structure, especially near the pressure peak region, thereby reducing internal film pressure. The reduction in pressure is attributed to two interrelated mechanisms: Increased flow resistance within the porous matrix, which alters the velocity profile and disrupts pressure development, and increased fluid loss through the porous wall, which lowers the effective lubricant thickness available for pressure generation in the film. Higher values of ${H_P}$​ (porous layer thickness) and ${R_P}$​ (capillary tube radius) amplify these effects by enhancing fluid diffusion pathways, weakening the hydrodynamic pressure field. Moreover, the porous design parameters strongly influence performance. Higher capillary tube radius or thicker porous pads reduce the system’s ability to retain lubricant within the load-bearing zone. Therefore, maintaining moderate porosity and fine pore dimensions is essential for sustaining higher pressure and enhancing the operational effectiveness of sinusoidal thrust bearings.

### Load-carrying capacity

**Fig. 5:**
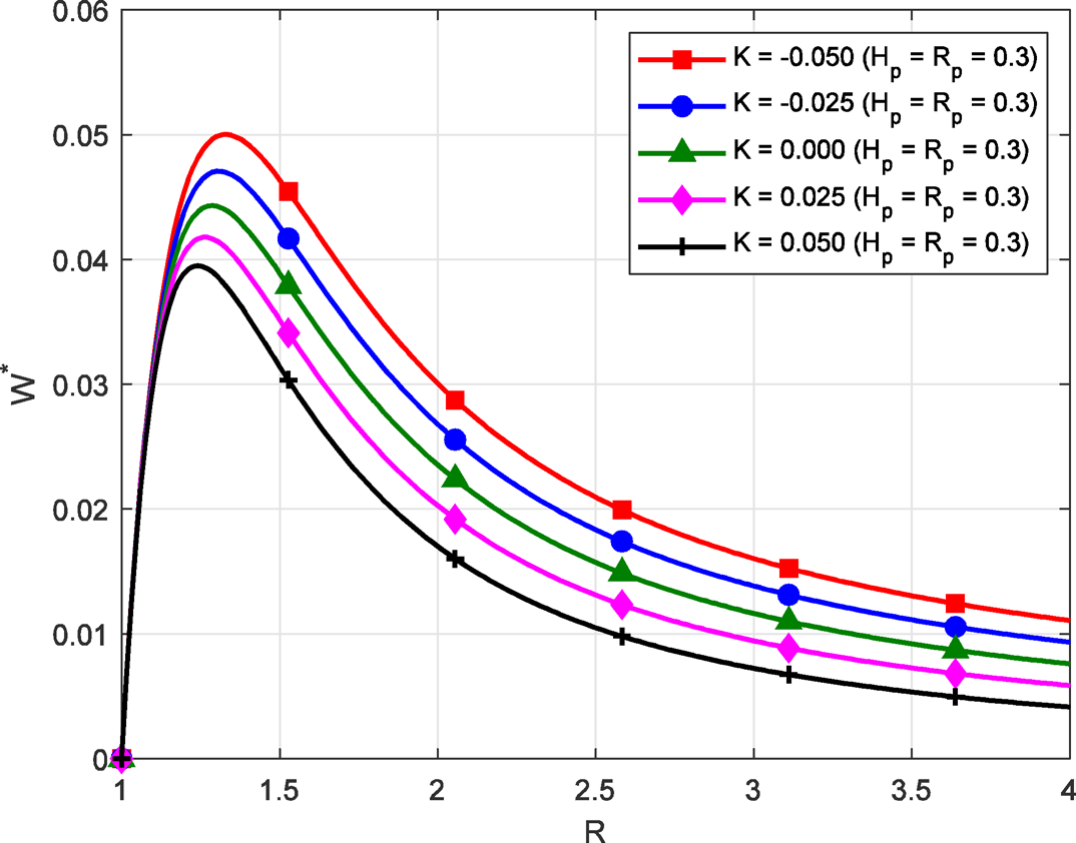
Load-carrying capacity $({W^*})$ versus inlet–outlet film ratio *R* for different values of *K* with ${H_P}$, ${R_P}$.

Figure 5 illustrates the variation of the non-dimensional load-carrying capacity as a function of the inlet–outlet film ratio R, for various values of the non-Newtonian parameter K, with the fixed porosity parameters.The load-carrying capacity shows a non-monotonic trend: it increases with R to a peak, then diminishes as R grows beyond this point.    . The phenomenon clearly indicates the presence of an optimal inlet-outlet ratio R at which the bearing achieves its highest load-carrying performance. The behavior is strongly influenced by the fluid’s shear characteristics. For dilatant fluids, the flow resistance increases with shear, which leads to stronger pressure gradients and greater internal pressure build-up. As a result, these fluids generate higher load-carrying capacities, especially near the optimal R region. On the other hand, pseudo-plastic fluids show lower resistance under shear, resulting in smoother flow but weaker pressure development and thus reduced load support.

The interplay between shear response and the inlet–outlet ratio governs how much fluid pressure can be sustained inside the sinusoidal film. At lower *R*, the inlet pressure dominates and promotes stronger internal pressure development. However, as *R* increases beyond the optimal point, the outlet pressure becomes relatively too small, and the fluid flow becomes overly unrestricted, leading to a drop in film pressure and reduced load capacity. Since ${H_P}$ and ${R_P}$​ remain constant, these effects are entirely attributed to the combined influence of *R* and *K*. Overall, the figure highlights that dilatant fluids provide better load-carrying performance due to stronger pressure buildup from their resistance to shear, while pseudo-plastic fluids are less effective in maintaining pressure. Selecting an appropriate combination of *R* and *K* is therefore critical in optimizing the performance of non-Newtonian lubricated thrust bearings.

**Fig. 6:**
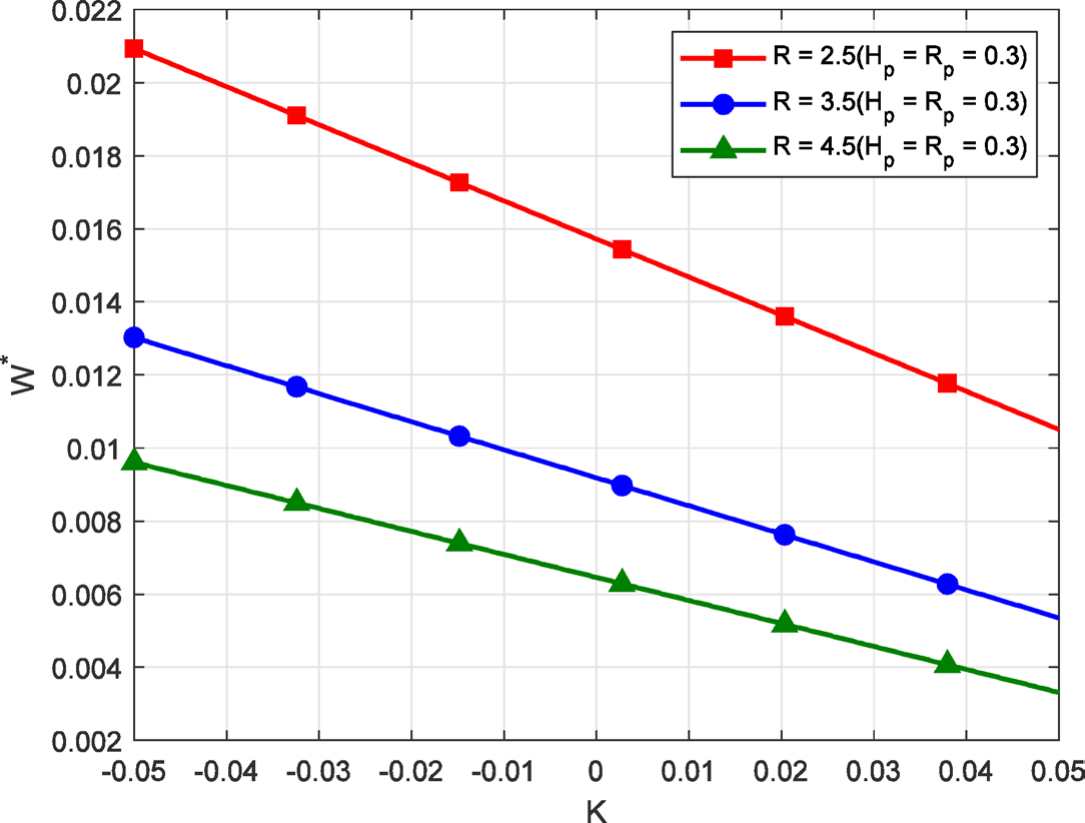
Load-carrying capacity $({W^*})$ vs. non-Newtonian parameter *K* for different values of *R* with ${H_P}$, ${R_P}$.

Figure 6 illustrate the variation of load-carrying capacity with the non-Newtonian parameter K, for various values of the inlet–outlet ratio R, while keeping the porous. In all cases, load carrying capacity decreases linearly as K increases. It reveals that shear-thickening fluids promote higher load support compared to shear-thinning fluids. From a physical perspective, when K is negative, the fluid exhibits higher resistance to deformation under increasing shear conditions. The behavior enhances internal flow resistance and pressure buildup within the lubricating film. Consequently, more pressure is retained inside the system, which contributes to a greater load-carrying response. As R increases, the value of load carrying capacity decreases for all K, consistent with previous observations. A higher R leads to more fluid escaping from the outlet, reducing the system’s ability to sustain pressure. The combined effect of a lower non-Newtonian parameter and a moderate inlet–outlet pressure ratio enhances film support efficiency, emphasizing the critical role of fluid rheology and load-carrying performance within porous thrust bearing systems.

**Fig. 7:**
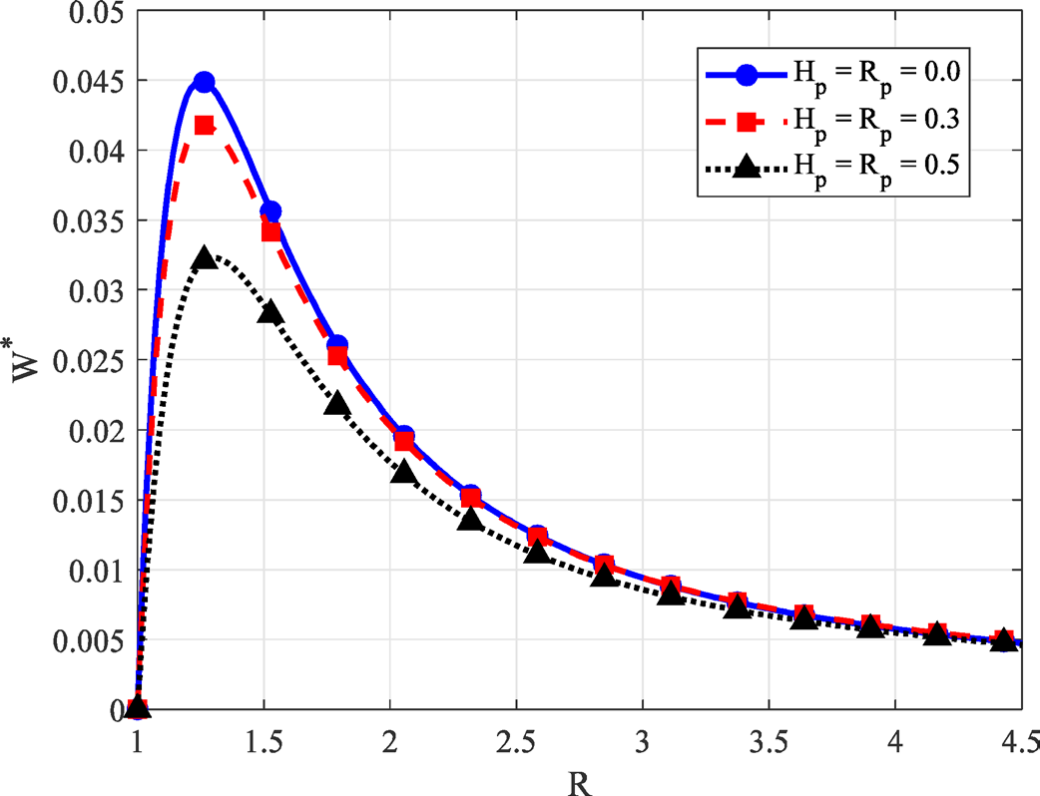
Load-carrying capacity $({W^*})$ versus inlet–outlet film ratio *R* for different values of ${H_p},\,{R_p}$ with *K*.

Figure 7 illustrates the variation in load-carrying capacity $({W^*})$ as a function of the inlet–outlet film ratio *R*, for different values of porous parameters ${H_P}={R_P}$​, while keeping the fluid property $K=0.025$ constant. Across all conditions, ${W^*}$ initially increases with *R*, reaches a peak, and then decreases as *R* continues to rise. The presence of a distinct maximum highlights an optimal film ratio where the bearing achieves its highest support performance. As the porous parameters ${H_P}$ and ${R_P}$ ​increase, a significant reduction in ${W^*}$ is observed throughout the range of *R*. Two physical mechanisms account for this decline. First, greater porosity promotes fluid penetration into the pad, reducing the effective film thickness that contributes to film generation. Second, higher resistance within the porous layer restricts internal flow, limiting the system’s ability to sustain a pressure gradient. When porosity is low, the fluid remains more confined within the lubricating film, allowing pressure to build effectively and maintain higher load capacity. In contrast, a more porous structure leads to increased fluid loss and diminished internal pressure, directly lowering the film’s load-supporting potential. An efficient porous thrust bearing must therefore balance pressure development and fluid retention. Moreover, the porous geometry and inlet–outlet ratio is essential to enhance hydrodynamic performance and ensure reliable load support under non-Newtonian lubrication.

### Volume flow rate

**Fig. 8:**
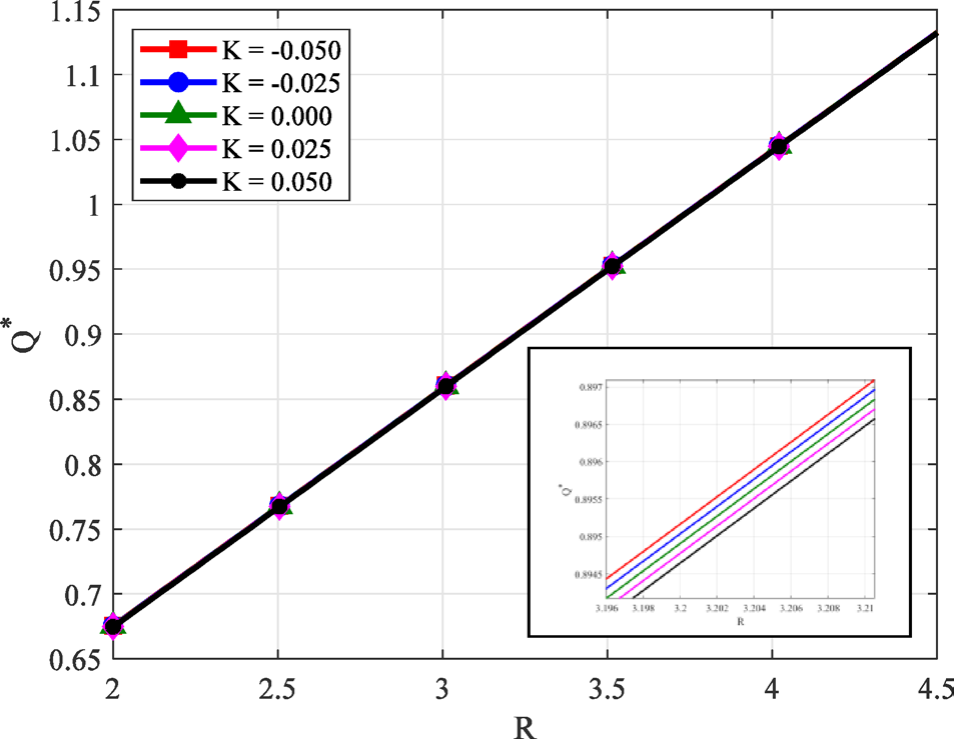
Variation of volume flow rate ${Q^*}$ along inlet–outlet film ratio *R* with ${H_P}$, ${R_P}$ for different values of *K*.

The graph in Fig. 8 illustrates the relationship between the non-dimensional volume flow rate $({Q^*})$ and the inlet–outlet film ratio *R* for various values of the non-Newtonian parameter *K*, with constant porous pad height $({H_P}=0.3)$ and capillary tube resistance ${R_P}=0.3$. The results clearly show that ${Q^*}$ increases almost linearly with increasing *R*, indicating enhanced fluid transport at higher pressure ratios. Notably, the flow rate curves for different *K* values are nearly overlapping, revealing that variations in the non-Newtonian parameter have a negligible impact on ${Q^*}$. It indicates that while *K* plays a critical role in determining the load-carrying capacity, it has minimal impact on overall flow rate under the specific conditions.

## Conclusion

This study examines the lubrication performance of sine film T-bearings with porous walls and considers the impact of n-N Rabinowitsch fluids. The investigation is conducted using a nonlinear n-N Reynolds equation that is developed from the RF model. Analytical formulations for load-carrying capacity, pressure distribution and volume flow rate are created by using the small perturbation technique. In comparison to Newtonian and pseudo-plastic lubricants, dilatant fluids consistently demonstrate superior performance by producing higher peak pressures, greater load-carrying capacities, and enhanced volume flow rates. These advantages are attributed to the shear-thickening nature of dilatant fluids, which promotes stronger resistance to deformation and allows significant pressure buildup within the converging regions of the film. Performance gains become especially prominent when the porous pad exhibits moderate thickness and the capillary tube remains low. Under these conditions, the lubricant is more effectively confined within the film, and the effects of shear are intensified, leading to better pressure development and fluid flow. However, as porosity increases through greater pad height or higher flow resistance, the ability to retain pressure diminishes. Fluid escapes into the porous structure more readily, reducing effective film thickness and weakening hydrodynamic support. Overall, the study emphasize that optimizing thrust bearing performance requires a delicate balance between the inlet–outlet film ratio, fluid behavior, and porous geometry. Dilatant lubricants, when paired with appropriately designed porous structures, offer a robust solution for maximizing load support and flow efficiency in high-shear, confined flow environments.

## Validation and future research

Although direct experimental or CFD validation is beyond the scope of the present study, the theoretical results were benchmarked against well-established limiting cases to verify the model’s reliability. The pressure distribution (Fig. 3) and load-carrying capacity behavior (Figs. 4 and 5) were compared with classical results by Lin^10,11^ under simplified conditions (e.g., Newtonian flow and non-porous walls), and showed strong agreement. These validations confirm that the current non-Newtonian model not only generalizes earlier formulations but also maintains consistency with foundational analytical solutions.

To address this, future work will involve:

- Developing CFD simulations that incorporate Rabinowitsch fluid rheology and porous media flow, enabling direct comparison with analytical predictions;
- Designing and fabricating experimental test rigs with porous pads to measure pressure profiles, film thickness, and flow behavior for various combinations of inlet–outlet pressure ratio $(R)$, porous pad thickness $({H_P})$, capillary resistance $({R_P})$, and non-Newtonian parameter $(K)$;
- Using the resulting CFD and experimental data to refine the theoretical model under more realistic operating conditions.

In addition to validation, several broader future research directions hold promise for advancing the field:

- Investigating other non-Newtonian fluid models (e.g., Bingham plastic, Carreau, or Casson fluids) to evaluate different rheological effects;
- Exploring the role of thermal gradients, material conductivity, and temperature-dependent fluid properties in performance prediction;
- Optimizing porous wall design through advanced manufacturing and tailored porosity distributions;
- Applying data-driven and machine learning techniques for predictive modeling and design optimization of thrust bearing systems.

By pursuing these directions, researchers can further improve the efficiency, robustness, and applicability of sine film thrust bearings. Such advancements have the potential to enable innovative, energy-efficient solutions across tribological, mechanical, and fluid engineering systems.

## Data Availability

All data generated or analysed during this study are included in this published article.

## List of symbols

${\bar {\tau }_{xy}}$: Stress tensor component
$\bar {k}$: Nonlinear component adjusting for RF effects
*K*: Dimensionless non-linear parameter
${\bar {\mu }_0}$: Viscosity of the Newtonian fluid
${w_{prs}}$: The flow velocity at the upper boundary of the porous layer
$\bar {u}$&$\bar {v}$: $\bar {x}$and $\bar {y}$directions velocity
*X*: Dimensionless coordinate axis
$$\bar {h}$$: Film height
${\bar {h}_0}$: Inlet film height
${\bar {h}_1}$: Outlet film height
*H*: Dimensionless film thickness
*R*: Dimensionless inlet-outlet film ratios
$\bar {w}$: Load-carrying capacity
${W^*}$: Dimensionless load carrying capacity
$\bar {q}$: Volume flow rate
${Q^*}$: Dimensionless volume flow rate
${\bar {v}_s}$: Sliding velocity in the x-direction
${\bar {B}_1}$: Bearing length in the x-direction
${\bar {B}_2}$: Bearing width perpendicular to the $\bar {x}\,\bar {z}$-plane
$$\bar {p}$$: Film pressure
${P^*}$: Dimensionless film pressure
$$\bar {t}$$: Response time
*p *: Pressure in the porous region
$\overline {{{H_p}}}$: Thickness of porous pad
${H_p}$: Dimensionless porous pad thickness
${r_c}$: Radius of capillary tube
${R_p}$: Non-dimensional radii of capillary tube
$${\phi _p}$$: Porosity coefficient
$\phi$: Fluid permeability in the porous region

## Acronyms

n-N: non-Newtonian
RF: Rabinowitsch fluid
T-bearing: Thrust bearing
